# Intense Near-Infrared Light-Emitting NaYF_4_:Nd,Yb-Based Nanophosphors for Luminescent Solar Concentrators

**DOI:** 10.3390/ma16083187

**Published:** 2023-04-18

**Authors:** A-Ra Hong, Seungyong Shin, Gumin Kang, Hyungduk Ko, Ho Seong Jang

**Affiliations:** 1 Materials Architecturing Research Center, Korea Institute of Science and Technology, 5 Hwarang-ro 14-gil, Seongbuk-gu, Seoul 02792, Republic of Korea; kongr.hongr@gmail.com (A.-R.H.); ljhbb0211@kist.re.kr (S.S.); 2 Nanophotonics Research Center, Korea Institute of Science and Technology, 5 Hwarang-ro 14-gil, Seongbuk-gu, Seoul 02792, Republic of Korea; guminkang@kist.re.kr (G.K.); kohd94@kist.re.kr (H.K.); 3 Division of Nano & Information Technology, KIST School, Korea University of Science and Technology (UST), Seoul 02792, Republic of Korea

**Keywords:** downshifting, nanophosphors, core/shell/shell, transparent polymer composite, luminescent solar concentrator

## Abstract

In this study, we synthesized NaYF_4_-based downshifting nanophosphors (DSNPs), and fabricated DSNP-polydimethylsiloxane (PDMS) composites. Nd^3+^ ions were doped into the core and shell to increase absorbance at 800 nm. Yb^3+^ ions were co-doped into the core to achieve intense near-infrared (NIR) luminescence. To further enhance the NIR luminescence, NaYF_4_:Nd,Yb/NaYF_4_:Nd/NaYF_4_ core/shell/shell (C/S/S) DSNPs were synthesized. The C/S/S DSNPs showed a 3.0-fold enhanced NIR emission at 978 nm compared with core DSNPs under 800 nm NIR light. The synthesized C/S/S DSNPs showed high thermal stability and photostability against the irradiation with ultraviolet light and NIR light. Moreover, for application as luminescent solar concentrators (LSCs), C/S/S DSNPs were incorporated into the PDMS polymer, and the DSNP-PDMS composite containing 0.25 wt% of C/S/S DSNP was fabricated. The DSNP-PDMS composite showed high transparency (average transmittance = 79.4% for the visible spectral range of 380–750 nm). This result demonstrates the applicability of the DSNP-PDMS composite in transparent photovoltaic modules.

## 1. Introduction

The development of advanced building-integrated photovoltaics (BIPVs) can address space constraint issues in high-rise buildings and meet their energy needs [1]. In the BIPVs, the utilization of luminescent solar concentrators (LSCs) enables the realization of semi-transparent photovoltaic (PV) modules for converting the facades of the urban building into energy generators [2]. LSCs are typically fabricated as waveguides consisting of transparent surfaces either coated with or matrices containing luminescent materials, such as organic dyes, quantum dots (QDs), or inorganic phosphors [3,4]. Although organic dyes have been used in the LSCs, they are hampered by poor stability and self-absorption loss due to their small Stokes shift [3]. Recently, QDs have been explored as luminescent materials in LSCs owing to their higher stability and wider absorption band width than organic dyes [5]. Nonetheless, QDs have small Stokes shifts and suffer from self-absorption loss [3]. To overcome this issue, large Stokes shift-emitting QDs have been developed where host QD nanocrystals were doped with transition metal ions, such as Mn^2+^, to emit long-wavelength visible light under short-wavelength ultraviolet (UV) light excitation [6,7].

On the other hand, lanthanide-doped inorganic phosphors are promising candidates for use in LSCs [8,9]. Compared to organic dyes and QDs, lanthanide-doped inorganic phosphors are more advantageous because they generally exhibit a large Stokes shift [9]. For example, de Boer et al. applied SrB_4_O_7_:Sm,Eu phosphors to an LSC film, where the SrB_4_O_7_:Sm,Eu phosphor showed an absorption band below 600 nm and an emission peak at 685 nm [9]. Liu et al. reported an LSC film consisting of a poly(methyl methacrylate) waveguide containing CaAlSiN_3_:Eu^2+^ phosphors with a Stokes shift of 112 nm [10]. In addition, Weber and Lambe reported that Nd^3+^-doped glass could be a luminescent medium because Nd^3+^-ions have strong absorption bands in the 500–900 nm range and an emission peak at 1060 nm, which is a wavelength well suited for use in silicon solar cells [11]. Thus, Nd^3+^-doped phosphors appear desirable for LSCs due to the amelioration of the above drawbacks that plague organic dyes and QDs [11]. However, micrometer-sized phosphors exhibit a scattering issue that increases non-emissive absorption and escape cone losses when these phosphors are utilized in LSCs [8]. This scattering issue can be addressed using nanophosphors. According to Do’s group, light scattering decreases as the phosphor size decreases, and nanophosphors smaller than 50 nm can lead to transparent nanophosphor-based matrix [12]. Thus, Nd^3+^-doped nanophosphors, smaller than 50 nm, can be applied to a transparent LSCs coupled with silicon solar cells.

Although Nd^3+^-doped nanophosphors have recently been reported, most of these studies focused on Nd^3+^-doped upconversion nanophosphors for bio-related applications [13,14,15,16]. In contrast, herein, we focus on Nd^3+^-doped downshifting nanophosphors (DSNPs) for transparent LSC applications. As described above, the Nd^3+^ ions exhibit an emission peak at 1060 nm in Nd^3+^-doped materials [11,16]. Additionally, the Nd^3+^ ions can be deployed as sensitizers to enhance the luminescence of nanophosphors [17,18]. In particular, near-infrared (NIR) emission can be enhanced by Nd^3+^–Yb^3+^ energy transfer [17]. This study prompted us to develop Nd^3+^-sensitized and Yb^3+^-activated DSNPs. In the current study, we synthesized NaYF_4_:Nd^3+^,Yb^3+^-based DSNPs with a core/shell/shell (C/S/S) structure for strong NIR emission. The DSNP core was coated with an Nd^3+^-doped active shell and a NaYF_4_ inert shell. In addition, we fabricated a transparent polydimethylsiloxane (PDMS) composite encapsulating NaYF_4_:Nd,Yb-based C/S/S DSNPs to investigate the feasibility of applying C/S/S DSNPs to LSCs.

## 2. Materials and Methods

For the syntheses of core, core/shell (C/S), and C/S/S DSNPs, YCl_3_∙6H_2_O (99.99%), NdCl_3_∙6H_2_O (99.9%), YbCl_3_∙6H_2_O (99.9%), NaOH (99.99%), NH_4_F (≥99.99%), 1-Octadecene (ODE, 90%, technical grade), and oleic acid (OA, 90%, technical grade) were purchased from Sigma-Aldrich (St. Louis, MO, USA).

We synthesized Nd^3+^ and Yb^3+^-doped NaYF_4_ core DSNPs using a co-precipitation method [19]. To synthesize the core DSNPs, YCl_3_·6H_2_O (0.9 − x mmol), NdCl_3_·6H_2_O (x mmol, x = 0.1, 0.2, 0.25, 0.3, 0.4 and 0.5), YbCl_3_·6H_2_O (0.1 mmol), 6 mL of OA, and 15 mL of ODE were heat-treated at 150 °C for 40 min. After cooling the reaction solution to room temperature, 10 mL of methanol (MeOH) solution comprising NaOH (2.5 mmol) and NH_4_F (4 mmol) was added to it, and the solution was stirred for 40 min. MeOH was removed, and core DSNPs were synthesized by heat treatment at 320 °C for 1 h. The core DSNPs were washed with MeOH, ethanol, and hexane, and then dispersed in 10 mL of chloroform.

To synthesize the C/S DSNPs, YCl_3_·6H_2_O (0.6 − x mmol), NdCl_3_·6H_2_O (x mmol, x = 0, 0.06, 0.12, 0.18, 0.24), 6 mL of OA, and 15 mL of ODE were loaded into a 3-neck flask and heat-treated at 150 °C. After the reaction solution was cooled to room temperature, core DSNPs and MeOH solution containing NaOH and NH_4_F were added to it. After removing MeOH, C/S DSNPs were synthesized by heat treatment at 320 °C for 1 h. The synthesized C/S DSNPs were washed in the same manner as the core DSNPs and dispersed in 10 mL of chloroform.

To synthesize C/S/S DSNPs, YCl_3_·6H_2_O (1 mmol), OA (6 mL), and ODE (15 mL) were mixed and heat-treated at 150 °C for 40 min. After the heat treatment, the reaction mixture was cooled to room temperature, followed by the addition of C/S DSNPs solution and MeOH solution containing NaOH (2.5 mmol) and NH_4_F (4 mmol) to it. The subsequent synthetic process was the same as that used to synthesize C/S DSNPs.

To prepare the PDMS-based LSC film, the C/S/S DSNP solution (0.5 mL) was mixed with SYLGARD 184 silicone elastomer (10 mL, Dow Chemical Company, Midland, MI, USA). The curing agent (1 mL) was then added to the mixture of the C/S/S DSNPs and silicone elastomer. Subsequently, the mixture was poured into a mold and baked at 80 °C for 1 h.

Transmission electron microscopy (TEM) images were obtained using a Tecnai F20 G^2^ transmission electron microscope (FEI Co., Hillsboro, OR, USA) at 200 kV. Energy-dispersive X-ray spectroscopy (EDS) was conducted using an FEI Talos F200X (FEI Co., Hillsboro, OR, USA) instrument at 200 kV. Structural analysis based on X-ray diffraction (XRD) was performed using a Bruker D8 Advance X-ray diffractometer with Cu-Kα radiation. Photoluminescence (PL) spectra were obtained using a PL/PLE-500 (PSI Trading Co., Ltd, Suwon, Republic of Korea) coupled with an 800 nm NIR diode laser. Time-resolved PL spectra were obtained by monitoring at 978 nm under pulsed irradiation with 800 nm NIR light using an InGaAs detector (IGA-030-TE2-H Photodetector, Dongwoo Optron Co., Ltd., Gwangju-si, Republic of Korea) and a monochromator (Monora323i, Dongwoo Optron Co., LTD., Republic of Korea). Absorption spectra were recorded using a Perkin-Elmer Lambda25 UV/vis spectrophotometer (Perkin-Elmer, Waltham, MA, USA). Transmittance spectra were obtained using a Shimadzu UV-3600 plus a UV-VIS-NIR spectrophotometer (Shimadzu, Kyoto, Japan).

## 3. Results and Discussion

**Figure 1:**
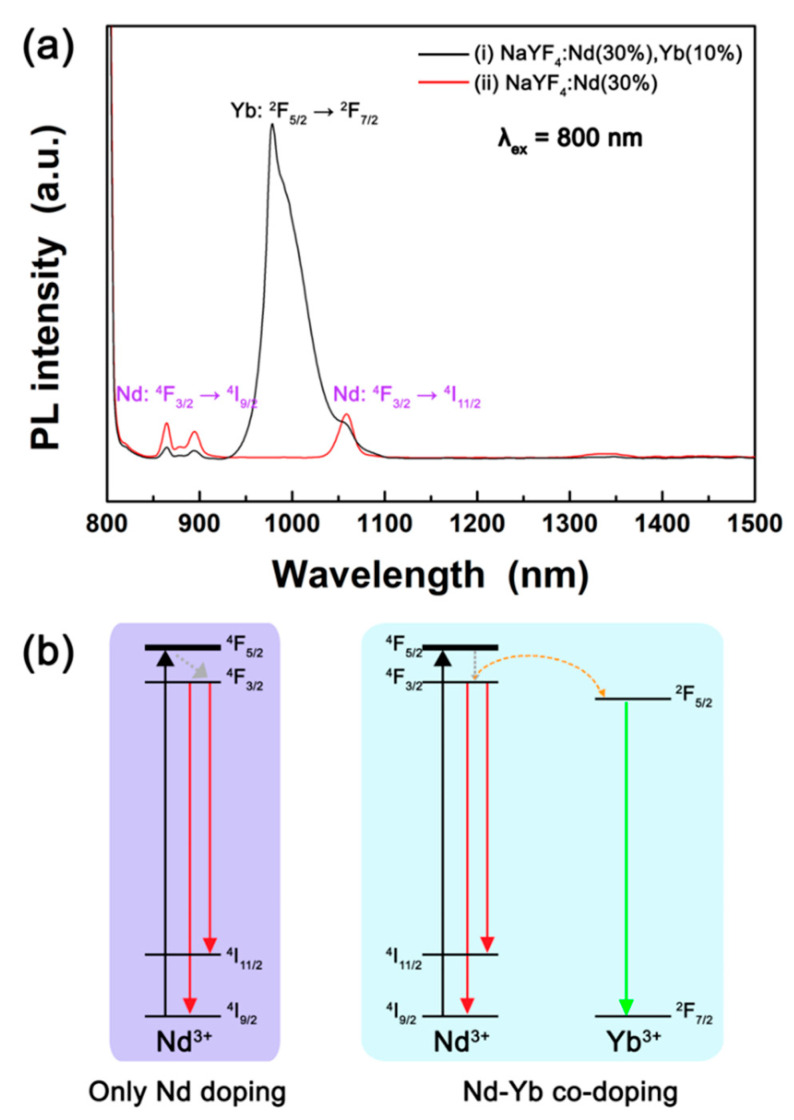
(**a**) PL spectra of (i) NaYF_4_:Nd (30%),Yb (10%) and (ii) NaYF_4_:Nd (30%) core DSNPs under 800 nm NIR light. (**b**) Schematic energy level diagram showing electronic transitions in Nd^3+^-doped and Nd^3+^ and Yb^3+^ co-doped DSNPs.

Figure 1 shows the PL spectra of NaYF_4_:Nd (30%),Yb (10%) and NaYF_4_:Nd (30%) core DSNPs under 800 nm NIR light. The NaYF_4_:Nd (30%),Yb (10%) core DSNPs exhibited strong Yb^3+^ emission band peaking at 978 nm and weak Nd^3+^ peaks at 862 nm and 1060 nm. In contrast, the NaYF_4_:Nd (30%) core DSNPs only showed Nd^3+^ emission peaks at 862 nm and 1060 nm. As shown in Figure 1a, the emission intensity of Yb^3+^ ions is much stronger than that of Nd^3+^ ions, indicating that the Nd^3+^ and Yb^3+^ co-doped DSNPs have the potential to achieve strong NIR-to-NIR emission. Figure 1b shows the energy level diagram of the Nd^3+^ and Yb^3+^ ions. When the NaYF_4_:Nd core DSNPs are irradiated with 800 nm NIR light, the energy is absorbed by the Nd^3+^ ions, resulting in ^4^I_9/2_ → ^4^F_5/2_ transition, and then the excited energy is relaxed to the ^4^F_3/2_ level [20]. As a result, Nd^3+^ shows PL peaks at 862 nm and 1060 nm owing to ^4^F_3/2_ → ^4^I_9/2_ and ^4^F_3/2_ → ^4^I_11/2_ electronic transitions, respectively [20]. In NaYF_4_:Nd,Yb core DSNPs, the energy absorbed by Nd^3+^ ions is transferred to the ^2^F_5/2_ level of the adjacent Yb^3+^ ions, resulting in a broad NIR emission band peaking at 978 nm due to the ^2^F_5/2_ → ^2^F_7/2_ transition [20]. Because NaYF_4_:Nd,Yb emits strong and broad emission under 800 nm NIR light, we adopted NaYF_4_:Nd,Yb as the core DSNP and optimized the Nd^3+^ concentration for further enhancement of the NIR-to-NIR emission.

**Figure 2:**
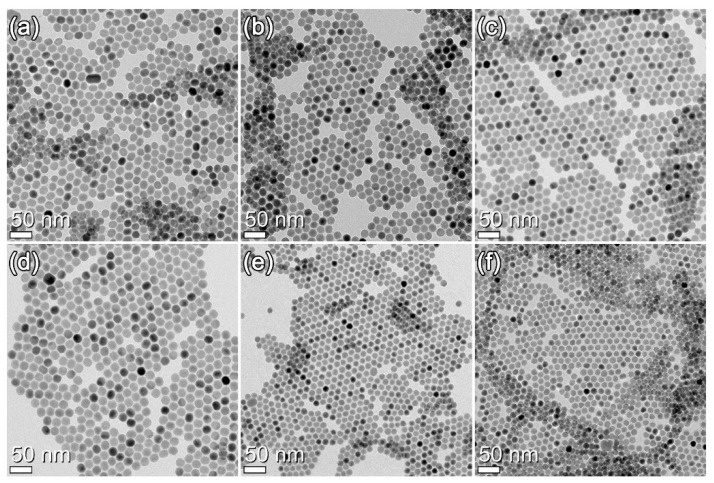
TEM images of NaYF_4_:Nd (x%),Yb (10%) core DSNPs with various Nd^3+^ concentrations of (**a**) 10%, (**b**) 20%, (**c**) 25%, (**d**) 30%, (**e**) 40%, and (**f**) 50%.

Figure 2 shows TEM images of NaYF_4_:Nd (x%),Yb (10%) core DSNPs with various Nd^3+^ concentrations. The sizes of NaYF_4_:Nd (10%),Yb (10%), NaYF_4_:Nd (20%),Yb (10%), NaYF_4_:Nd (25%),Yb (10%), NaYF_4_:Nd (30%),Yb (10%), NaYF_4_:Nd (40%),Yb (10%), and NaYF_4_:Nd (50%),Yb (10%) core DSNPs were found to be 20.8 ± 0.9 nm, 19.6 ± 0.7 nm, 19.1 ± 0.7 nm, 20.0 ± 0.9 nm, 14.1 ± 0.8 nm, and 13.4 ± 0.9 nm (average size ± standard deviation), respectively (Figure S1). Figure 2 and Figure S1 showed that DSNPs with uniform size and morphology were synthesized. The size of the synthesized core DSNPs decreased with increasing Nd^3+^ concentrations. This result suggests that some of the Y^3+^ ions (r = 1.159 Å) in the NaYF_4_ host lattice were replaced with Nd^3+^ ions (r = 1.249 Å) because doping with lanthanide ions larger than the Y^3+^ ion leads to a decrease in particle size [21]. The XRD patterns of the synthesized core DSNPs are shown in Figure S2. The XRD patterns exhibited that the core DSNPs with a single hexagonal phase were synthesized without impurities even when the concentration of Nd^3+^ ions in the core increased to 50%. In addition, the XRD patterns show that the (110) and (101) diffraction peaks observed at approximately 29° and 30° shifted to lower angles as the concentration of Nd^3+^ ions increased. The XRD peak shift is due to the replacement of Y^3+^ ions with Nd^3+^ ions, which are comparatively larger than Y^3+^ ions [22]. Figure S3 shows EDS map images and EDS spectrum obtained for the NaYF_4_:Nd (30%),Yb (10%) core DSNPs. Nd Lα, Yb Lα, and Y Kα peaks were observed in the EDS spectrum, and the EDS maps for the dopants Nd and Yb overlapped with the Y Kα map, confirming that NaYF_4_ was co-doped with Nd^3+^ and Yb^3+^ ions.

**Figure 3:**
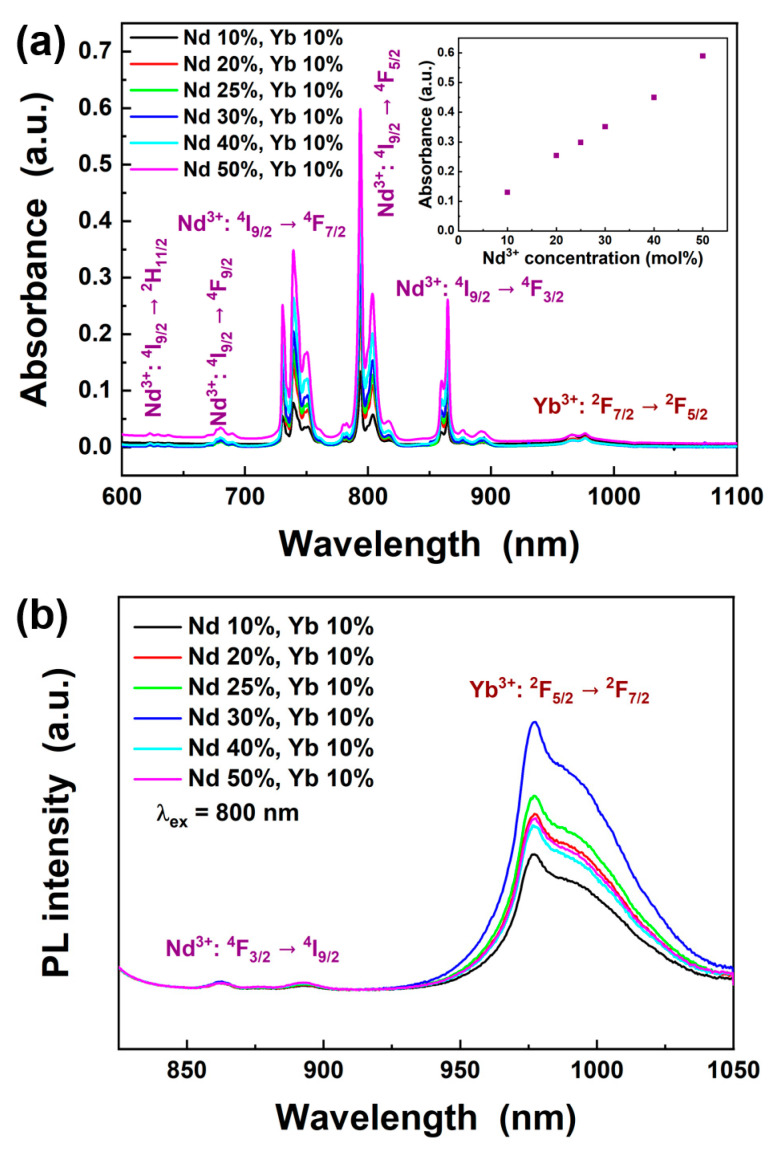
(**a**) Absorption and (**b**) PL spectra of NaYF_4_:Nd (x%),Yb (10%) core DSNPs with various Nd^3+^ concentrations (x = 10, 20, 25, 30, 40, and 50%) under 800 nm NIR light. Inset shows absorbance values at 794 nm, depending on the Nd^3+^ concentrations.

Figure 3a shows the absorption spectra of the NaYF_4_:Nd (x%),Yb (10%) core DSNPs with various Nd^3+^ concentrations. The absorption peaks are attributed to the electronic transitions of ^4^I_9/2_ → ^2^H_11/2_ (623 nm), ^4^I_9/2_ → ^4^F_9/2_ (680 nm), ^4^I_9/2_ → ^4^F_7/2_ (740 nm), ^4^I_9/2_ → ^4^F_5/2_ (794 nm), and ^4^I_9/2_ → ^4^F_3/2_ (865 nm) [23,24,25,26]. In contrast, the absorption peak observed at 976 nm is due to the ^2^F_7/2_ → ^2^F_5/2_ electronic transition of Yb^3+^ ions [20,23,24]. The inset of Figure 3a shows that the absorbance of the NaYF_4_:Nd,Yb core DSNPs at 794 nm systematically increased as the Nd^3+^ concentration increased. Figure 3b illustrates the PL spectra of NaYF_4_:Nd (x%),Yb (10%) core DSNPs with various Nd^3+^ concentrations. Characteristic emission peaks of Yb^3+^ and Nd^3+^ were observed at 978 nm and 862 nm, respectively. The emission intensity of Yb^3+^ increased as the concentration of Nd^3+^ in the core increased from 10% to 30%, whereas the emission intensity of Yb^3+^ decreased when the Nd^3+^ concentration was higher than 30%. Within the synthetic conditions applied in this study, NaYF_4_:Nd (30%),Yb (10%) showed the highest PL intensity under 800 nm excitation.

**Figure 4:**
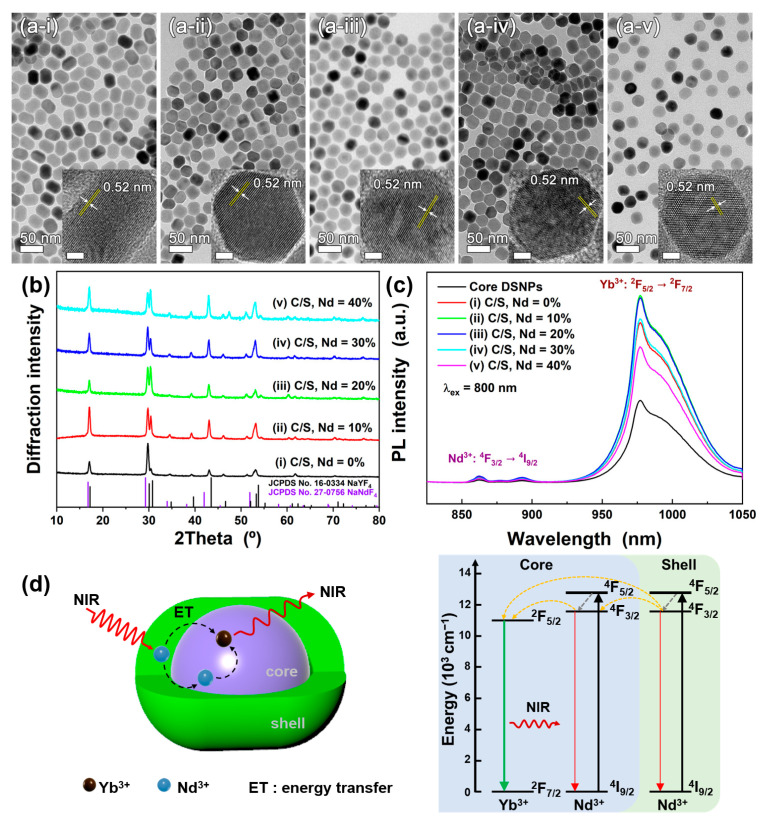
(**a**) TEM and HR-TEM images, (**b**) XRD patterns, and (**c**) PL spectra of NaYF_4_:Nd (30%),Yb (10%)/NaYF_4_:Nd (x%) C/S DSNPs with various Nd^3+^ concentrations [x = (**i**) 0%, (**ii**) 10%, (**iii**) 20%, (**iv**) 30%, and (**v**) 40%]. (**d**) Schematic illustration showing the core/shell structure of the NaYF_4_:Nd,Yb/NaYF_4_:Nd DSNP (left) and schematic energy level diagram showing the energy transfer from Nd^3+^ to Yb^3+^ in the C/S DSNPs (right). Scale bars in the insets of (**a**) indicate 5 nm.

It is well known that the efficiency of nanophosphors is sensitive to surface defects [27,28]. Passivation of the surface of nanophosphors is an efficient way to improve their luminescent properties [29]. Thus, the NaYF_4_:Nd (30%),Yb (10%)/NaYF_4_ C/S DSNPs were synthesized to enhance the emission intensity of Yb^3+^ ions at 978 nm under 800 nm irradiation. Previously, Vetrone et al. reported that the active shell, where the shell was doped with sensitizer ions, was beneficial for further PL enhancement of nanophosphors [30]. Thus, the NaYF_4_ shell was doped with Nd^3+^ ions, and the C/S DSNPs could absorb more 800 nm NIR light than the core DSNPs. Figure 4 shows the TEM images of NaYF_4_:Nd (30%),Yb (10%)/NaYF_4_:Nd (x%) C/S DSNPs. The concentration of Nd^3+^ ion in the shell was adjusted to 0%, 10%, 20%, 30%, and 40%, and the corresponding C/S DSNPs sizes were measured to be 34.3 nm ± 1.2 nm × 26.7 nm ± 1.2 nm, 32.3 nm ± 1.0 nm × 26.2 nm ± 1.2 nm, 27.5 nm ± 1.2 nm, 26.3 nm ± 1.2, 26.2 nm ± 1.4 nm, respectively. The TEM results showed that NaYF_4_:Nd (30%),Yb (10%)/NaYF_4_:Nd (x%) C/S DSNPs with uniform sizes were synthesized. High resolution (HR)-TEM images of the C/S DSNPs are shown in Figure 4a insets. The HR-TEM images show that the NaYF_4_:Nd (30%),Yb (10%)/NaYF_4_:Nd (x%) C/S DSNPs exhibited high crystallinity and a hexagonal structure, judging from clear lattice fringes and the measured lattice spacing (0.52 nm), which coincides with *d*-spacing between the (10$\overset{-}{1}$0) planes of hexagonal NaYF_4_ [31]. The crystal structures of the NaYF_4_:Nd (30%),Yb (10%)/NaYF_4_:Nd (x%) C/S DSNPs were also investigated using the XRD patterns (Figure 4b). The XRD patterns show that the NaYF_4_:Nd (30%),Yb (10%)/NaYF_4_:Nd (x%) C/S DSNPs exhibited a single hexagonal phase without any impurity.

Figure 4c shows PL spectra of the NaYF_4_:Nd (30%),Yb (10%) core and NaYF_4_:Nd (30%),Yb (10%)/NaYF_4_:Nd (x%) C/S DSNPs under 800 nm NIR light. After the shell formation on the NaYF_4_:Nd (30%),Yb (10%) core, the C/S DSNPs showed higher PL intensity than the core DSNPs. The Yb^3+^ emission intensity of the C/S DSNPs was further enhanced by doping the shell with Nd^3+^ ions. In the PL spectra, we observed a weak Nd^3+^ emission peak at 862 nm (^4^F_3/2_ → ^4^I_9/2_) and a strong Yb^3+^ emission band at 978 nm (^2^F_5/2_ → ^2^F_7/2_) [15,20]. When the shell was doped with 10% Nd^3+^ ions, the NaYF_4_:Nd (30%),Yb (10%)/NaYF_4_:Nd (10%) C/S DSNPs exhibited the highest emission intensity. In contrast, when the Nd^3+^ concentration in the shell was higher than 10%, the Yb^3+^ emission intensity decreased as the Nd^3+^ concentration increased. Figure 4d shows a schematic illustration of the C/S DSNP and an energy level diagram of Yb^3+^ and Nd^3+^. Further enhancement of the Yb^3+^ emission intensity can be explained by energy transfer from Nd^3+^ in the shell to Nd^3+^/Yb^3+^ in the core [20,32]. When the C/S DSNPs are illuminated with 800 nm NIR light, it is absorbed by Nd^3+^ ions in the shell in addition to those in the core, and the excited energy is transferred to the Nd^3+^ ions in the core, followed by energy transfer from Nd^3+^ to Yb^3+^. As the concentration of Nd^3+^ ions increases in the shell, the energy loss can increase due to energy migration to the surface [28,33]. Consequently, the emission from the NaYF_4_:Nd (30%),Yb (10%)/NaYF_4_:Nd (x%) C/S DSNPs decreased when the concentration of Nd^3+^ ions in the shell was greater than 10% (Figure 4c).

**Scheme 1:**
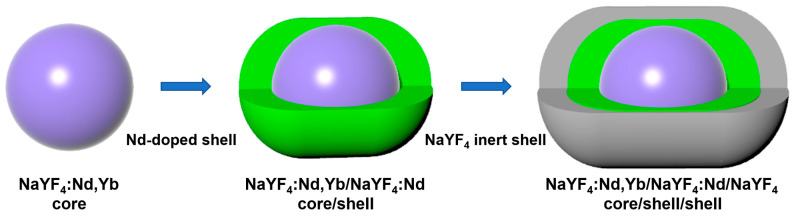
Schematic illustration showing the formation of core/shell/shell-structured DSNPs.

**Figure 5:**
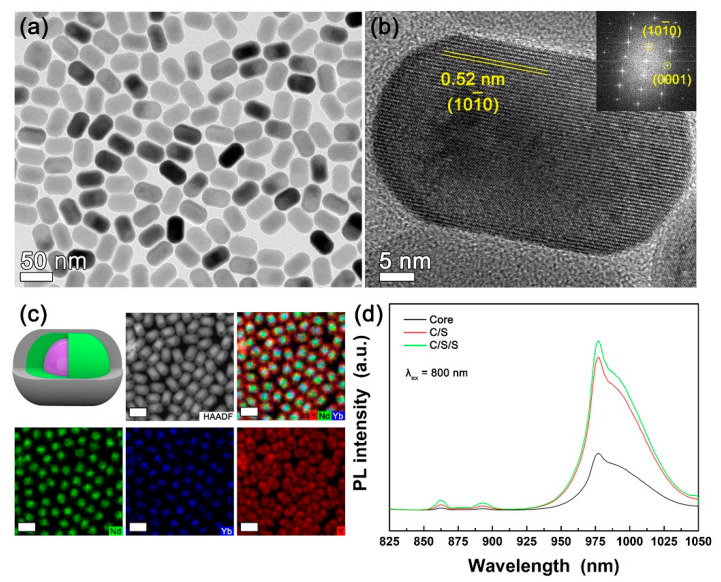
(**a**) TEM image and (**b**) HR-TEM image of NaYF_4_:Nd (30%),Yb (10%)/NaYF_4_:Nd (10%)/NaYF_4_ C/S/S DSNPs. The inset of (**b**) is an FFT pattern for the HR-TEM image shown in (**b**). (**c**) Schematic illustration showing the C/S/S-structured DSNP, HAADF-STEM image, and EDS map images (merged image, Nd Lα (green), Yb Lα (blue), and Y Kα (red) maps) of NaYF_4_:Nd (30%),Yb (10%)/NaYF_4_:Nd (10%)/NaYF_4_ C/S/S DSNPs. Scale bars in the (**c**) indicate 50 nm. (**d**) PL spectra of NaYF_4_:Nd (30%),Yb (10%) core, NaYF_4_:Nd (30%), Yb (10%)/NaYF_4_:Nd(10%) C/S, and NaYF_4_:Nd (30%),Yb (10%)/NaYF_4_:Nd (10%)/NaYF_4_ C/S/S DSNPs under 800 nm NIR light.

Because Nd^3+^ ions doped in the shell are affected by surface defects, the second shell was formed around the NaYF_4_:Nd (30%),Yb (10%)/NaYF_4_:Nd (10%) C/S DSNPs. As shown in Scheme 1, NaYF_4_:Nd (10%) shell was grown on the NaYF_4_:Nd (30%),Yb (10%) core DSNPs followed by the formation of NaYF_4_ shell. Since the crystal structure of NaYF_4_ is hexagonal, anisotropic shell growth on the NaYF_4_-based nanoparticles can occur [34,35]. Faster growth rate along [0001] direction rather than other crystallographic axes yields rod-like nanoparticles [35]. After growth of the NaYF_4_:Nd (10%) first shell on the core, the DSNPs showed rod-like morphology where the length along the [0001] direction was slightly larger than that along short axis. After the formation of the second shell on the C/S DSNPs, particle size increased and the C/S/S DSNPs apparently exhibits a rod shape. Figure 5a shows the TEM image of NaYF_4_:Nd (30%),Yb (10%)/NaYF_4_:Nd (10%)/NaYF_4_ C/S/S DSNPs. The NaYF_4_:Nd (30%),Yb (10%)/NaYF_4_:Nd (10%)/NaYF_4_ C/S/S DSNPs showed a uniform rod shape with the size of 45.9 nm ± 1.4 nm × 29.9 nm ± 1.3 nm. As shown in Figure S4, NaYF_4_:Nd (30%),Yb (10%)/NaYF_4_:Nd (10%)/NaYF_4_ C/S/S DSNPs with a single hexagonal phase were successfully synthesized without impurities. Figure 5b shows the HR-TEM image and the corresponding fast Fourier transformation (FFT) pattern of NaYF_4_:Nd (30%),Yb (10%)/NaYF_4_:Nd (10%)/NaYF_4_ C/S/S DSNPs. The NaYF_4_:Nd (30%),Yb (10%)/NaYF_4_:Nd (10%)/NaYF_4_ C/S/S DSNPs exhibited clear lattice fringes with a lattice spacing of 0.52 nm, which coincides with *d*-spacing between the (10$\overset{-}{1}$0) planes of hexagonal NaYF_4_. This result indicates that highly crystalline hexagonal-structured NaYF_4_:Nd (30%),Yb (10%)/NaYF_4_:Nd (10%)/NaYF_4_ C/S/S DSNPs were synthesized. Figure 5c shows a schematic illustration and EDS map images of the NaYF_4_:Nd (30%),Yb (10%)/NaYF_4_:Nd (10%)/NaYF_4_ C/S/S DSNPs. In the high-angle annular dark-field scanning TEM (HAADF-STEM) image, the core-shell structure in the DSNPs was roughly observed by brightness contrast. However, the C/S/S structure was not observed. Therefore, EDS analysis was conducted to investigate the C/S/S structure of DSNPs. The EDS map images show that Yb^3+^ and Nd^3+^ ions were present in the inner region of NaYF_4_:Nd (30%),Yb (10%)/NaYF_4_:Nd (10%)/NaYF_4_ C/S/S DSNPs, whereas Y^3+^ ions were present in all of the regions. A comparison of the Nd Lα and Yb Lα maps revealed that Nd^3+^ was observed in a wider area than Yb^3+^ doped in the core, indicating Nd^3+^ doping of the first shell. Magnified EDS maps also directly show the formation of core/shell/shell structure (Figure S5). Consequently, EDS analysis confirmed the successful synthesis of the C/S/S-structured DSNPs. Figure 5d shows the PL spectra of the NaYF_4_:Nd (30%),Yb (10%) core, NaYF_4_:Nd (30%),Yb (10%)/NaYF_4_:Nd (10%) C/S, and NaYF_4_:Nd (30%),Yb (10%)/NaYF_4_:Nd (10%)/NaYF_4_ C/S/S DSNPs under 800 nm NIR light. The NaYF_4_:Nd (30%),Yb (10%)/NaYF_4_:Nd (10%)/NaYF_4_ C/S/S DSNPs showed 3.0 times higher Yb^3+^ emission intensity than the core DSNPs. Moreover, the NaYF_4_:Nd (30%),Yb (10%)/NaYF_4_:Nd (10%)/NaYF_4_ C/S/S DSNPs exhibited 10% higher PL intensity than the C/S DSNPs.

When we investigated time-resolved PL properties of the NaYF_4_:Nd (30%),Yb (10%)-based DSNPs, PL lifetime of the DSNPs increased as the shell was grown on the core (Figure S6). The PL lifetimes of the DSNPs could be obtained by fitting the time-resolved PL profiles using an exponential decay function [36], and in our study, the time-resolved PL profiles were well fitted with bi-exponential and single exponential decay functions (Figure S6). The PL lifetime of the DSNPs increased from 611 μs for NaYF_4_:Nd (30%),Yb (10%) core DSNPs to 803 μs for NaYF_4_:Nd (30%),Yb (10%)/NaYF_4_:Nd (10%) C/S DSNPs. The NaYF_4_:Nd (30%),Yb (10%)/NaYF_4_:Nd (10%)/NaYF_4_ C/S/S DSNPs showed the longest PL lifetime (816 μs) among the core, C/S, and C/S/S DSNPs. The increase in the PL lifetime in the NaYF_4_:Nd (30%),Yb (10%)-based DSNPs indicates that the luminescence quenching is inhibited by the reduction of non-radiative pathway after shell formation [37]. Additionally, we investigated the stability of the NaYF_4_:Nd (30%),Yb (10%)/NaYF_4_:Nd (10%)/NaYF_4_ C/S/S DSNPs. As shown in Figure S7, the synthesized NaYF_4_:Nd (30%),Yb (10%)/NaYF_4_:Nd (10%)/NaYF_4_ C/S/S DSNPs showed high photostability and thermal stability. The C/S/S DSNPs exhibited stable PL properties against continuous irradiation of NIR light and ultraviolet light, respectively (Figure S7a–c). In addition, the C/S/S DSNPs showed stable PL characteristics after heat treatment at 100 °C for 1 h. These results indicate that the C/S/S DSNPs are beneficial for their practical application.

**Figure 6:**
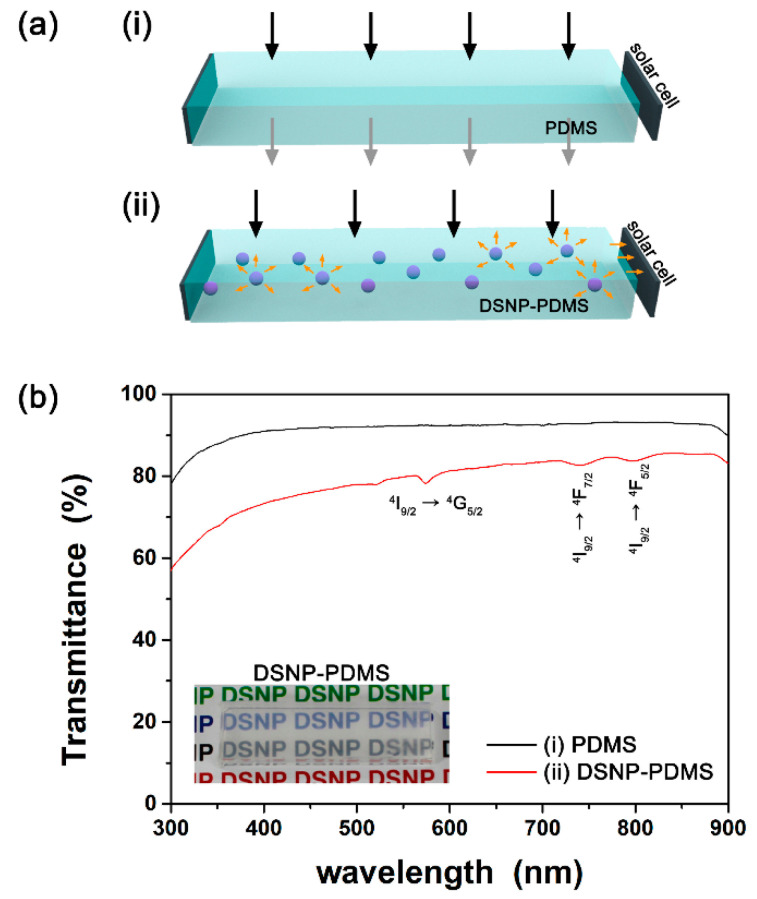
(**a**) Schematic illustration of (**i**) bare PDMS- and (**ii**) DSNP-PDMS composite-coupled solar cells. (**b**) Transmittance spectra of (**i**) the bare PDMS polymer and (**ii**) the C/S/S DSNP-PDMS composite. Inset shows a photograph of the C/S/S DSNP-PDMS composite.

To investigate the feasibility of LSC applications of the NaYF_4_:Nd (30%), Yb (10%)/NaYF_4_:Nd (10%)/NaYF_4_ C/S/S DSNPs, we fabricated NaYF_4_:Nd (30%),Yb (10%)/NaYF_4_:Nd (10%)/NaYF_4_ C/S/S DSNP-PDMS composites. Figure 6a shows a schematic illustration of bare PDMS- and DSNP-PDMS composite-coupled solar cells. In a bare PDMS-coupled solar cell, incident sunlight directly passes through the PDMS polymer so that most sunlight cannot reach the solar cell. In contrast, in the DSNP-PDMS composite-coupled solar cell, NIR photons of incident sunlight are absorbed by the DSNPs, and the DSNPs emit NIR light in all directions. The emitted light is directed to the silicon solar cell attached to the edge of the DSNP-PDMS composite, which can result in electricity generation from the silicon solar cell. Figure 6b shows the transmittance spectra of the bare PDMS and the C/S/S DSNP-PDMS composite. The average transmittance values of the bare PDMS and the C/S/S DSNP-PDMS composite in the visible region (λ = 380–750 nm) were 92.1 and 79.4%, respectively. Although the transmittance of the C/S/S DSNP-PDMS composite was lower than that of the bare PDMS polymer, it was still highly transparent, as shown in the inset of Figure 6b. Therefore, we believe that the C/S/S DSNP-PDMS composite can be applied to transparent PV modules. Additionally, in the transmittance spectrum of the C/S/S DSNP-PDMS composite, peaks were observed at 574, 740, and 794 nm, which were attributed to the light absorption via ^4^I_9/2_ → ^4^G_5/2_, ^4^I_9/2_ → ^4^F_7/2_, and ^4^I_9/2_ → ^4^F_5/2_ transitions of Nd^3+^ ions in the NaYF_4_:Nd (30%),Yb (10%)/NaYF_4_:Nd (10%)/NaYF_4_ C/S/S DSNPs, indicating the presence of NaYF_4_:Nd (30%),Yb (10%)/NaYF_4_:Nd (10%)/NaYF_4_ C/S/S DSNPs in the PDMS composite [25].

Figure S8a shows the current density versus voltage curves of the bare PDMS- and C/S/S DSNP-PDMS composite-coupled silicon solar cells. The C/S/S DSNP-PDMS composite-coupled silicon solar cell showed increased short-circuit current density compared with the bare PDMS-coupled silicon solar cell. The efficiencies of the bare PDMS- and the C/S/S DSNP-PDMS composite-coupled silicon solar cells were measured to be 0.92 and 1.96%, respectively, under Air Mass (AM) 1.5G illumination using a solar simulator. Since the transmittance of the C/S/S DSNP-PDMS composite is lower than the bare PDMS, the light scattering effect may contribute to the increase in solar cell efficiency. Since the absorption of the C/S/S DSNP-PDMS composite was low (Figure 6b), the contribution of light scattering to the increase in solar cell efficiency seems to be larger than that of the luminescence from the C/S/S DSNPs in the DSNP-PDMS composite to the increase in solar cell efficiency. However, when the C/S/S DSNP-PDMS composite was excited with weak 800 nm NIR light (1 mW), it showed clear emission band in the NIR spectral region (Figure S8b). Thus, it seems that both light scattering and luminescence due to the C/S/S DSNPs in the DSNP-PDMS composite contribute to the increase in solar cell efficiency, although the contribution of the luminescence from the C/S/S DSNPs may be low. Since the Nd^3+^ ions exhibit strong absorption peaks in NIR spectral region (Figure 3a), the absorption of the C/S/S DSNP-PDMS composite in the NIR spectral region can be enhanced after further optimization of the C/S/S DSNP-PDMS composite preparation. The transmittance of the C/S/S DSNP-PDMS composite can be further increased via further optimization of the DSNP-PDMS composite preparation. The contribution of the luminescence from the C/S/S DSNPs in the DSNP-PDMS composite will then increase, and the light scattering effect will decrease for the increase in solar cell efficiency. The optimized DSNP-PDMS composites can be suitable as LSCs for transparent PV modules.

## 4. Conclusions

We successfully synthesized NaYF_4_:Nd,Yb/NaYF_4_:Nd/NaYF_4_ C/S/S DSNPs that absorb 800 nm NIR light and emit broad-band NIR light peaking at 978 nm. The synthesized NaYF_4_:Nd (30%),Yb (10%)/NaYF_4_:Nd (10%)/NaYF_4_ C/S/S DSNPs exhibited a uniform rod shape and a single hexagonal phase. The EDS analysis confirmed the formation of the C/S/S structure. The Nd^3+^ ions doped in the core and shell can effectively absorb 800 nm NIR light, and the absorbed energy is transferred from the Nd^3+^ ions to the Yb^3+^ ions, resulting in a strong and broad NIR emission from the Yb^3+^ ions in the core. As a result, the luminescence intensity of the C/S/S DSNPs was significantly higher than that of the core DSNPs. The C/S/S DSNPs also exhibited high thermal stability and photostability. A transparent composite film was fabricated by incorporating C/S/S DSNPs into the PDMS polymer. This study shows that NaYF_4_:Nd,Yb-based C/S/S DSNPs can be used in LSCs and have potential applications to transparent PV modules.

## Data Availability

Data are contained within the article.

## Supplementary Materials

The following supporting information can be downloaded at: https://www.mdpi.com/article/10.3390/ma16083187/s1, Figure S1: Size distribution of NaYF_4_:Nd (x%),Yb (10%) core DSNPs with various Nd^3+^ concentrations; Figure S2: XRD patterns of NaYF_4_:Nd (x%),Yb (10%) core DSNPs with various Nd^3+^ concentrations; Figure S3: HAADF-STEM image and EDS map images of NaYF_4_:Nd (30%),Yb (10%) core DSNPs; Figure S4: XRD patterns of NaYF_4_:Nd (30%),Yb (10%)/NaYF_4_:Nd (10%)/NaYF_4_ C/S/S DSNPs; Figure S5: EDS map images of NaYF_4_:Nd (30%),Yb (10%)/NaYF_4_:Nd (10%)/NaYF_4_ C/S/S DSNPs; Figure S6: Time-resolved PL profiles of NaYF_4_:Nd (30%),Yb (10%)-based core, C/S, and C/S/S DSNPs [38]; Figure S7: Photo- and thermal stabilities of NaYF_4_:Nd (30%),Yb (10%)/NaYF_4_:Nd (10%)/NaYF_4_ C/S/S DSNPs; Figure S8: (a) Current density versus voltage curves of (i) bare PDMS- and (ii) C/S/S DSNP-PDMS composite-coupled silicon solar cells. (b) PL spectrum of the C/S/S DSNP-PDMS composite under excitation with 800 nm NIR light (1 mW).
