# Supplementary material for: Intense Near-Infrared Light-Emitting NaYF4:Nd,Yb-Based Nanophosphors for Luminescent Solar Concentrators
Source: Materials (Basel). 2023 Apr 18;16(8):3187. doi: 10.3390/ma16083187 (PMC10145680; doi:10.3390/ma16083187)
Supplement: Supplementary file 1 [file materials-16-03187-s001.zip › materials-2241861-SI.pdf]

materials

Supplementary Materials

Intense Near-Infrared Light-Emitting $NaYF_4$:Nd,Yb-Based Nanophosphors for Luminescent Solar Concentrators

A-Ra Hong [1], Seungyong Shin [1], Gumin Kang [2], Hyungduk Ko [2], and Ho Seong Jang [1,3,*]

[1] Materials Architecturing Research Center, Korea Institute of Science and Technology, 5, Hwarang-ro 14-gil, Seongbuk-gu, Seoul 02792, Republic of Korea; kongr.hongr@gmail.com(A.-R.H.); ljhbb0211@kist.re.kr (S.S.)
[2] Nanophotonics Research Center, Korea Institute of Science and Technology, 5, Hwarang-ro 14-gil, Seongbuk-gu, Seoul 02792, Republic of Korea; guminkang@kist.re.kr (G.K.); kohd94@kist.re.kr (H.K.)
[3] Division of Nano & Information Technology, KIST School, Korea University of Science and Technology (UST), Seoul 02792, Republic of Korea
* Correspondence: msekorea@kist.re.kr; Tel.: +82-2-958-5263

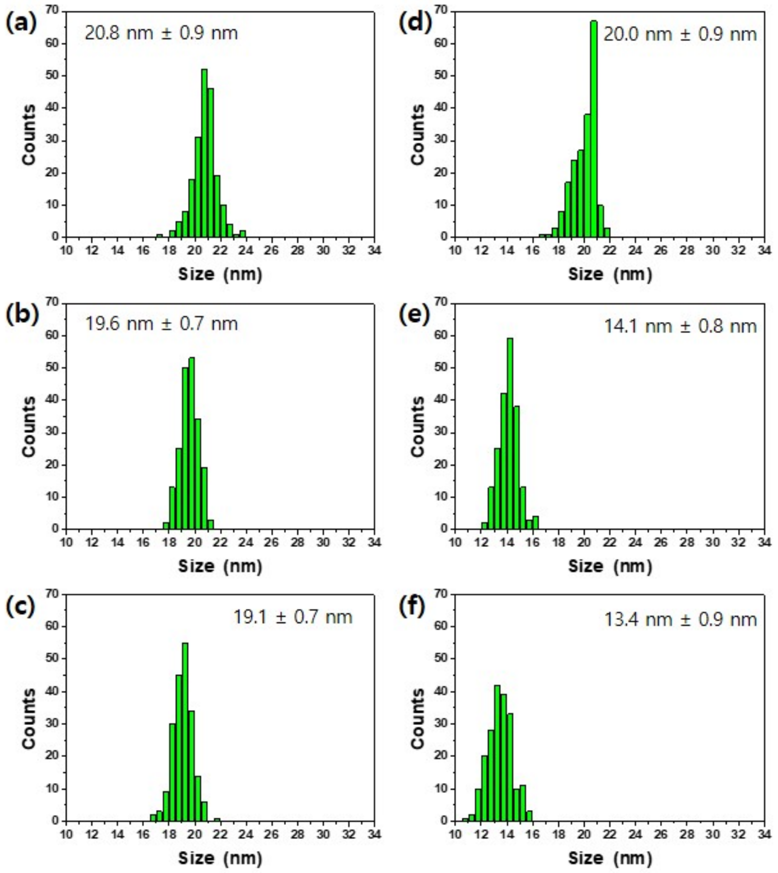

Figure S1. Size distributions of $NaYF_4$:Nd(x%),Yb(10%) core DSNPs with various Nd^{3+} concentrations [x = (a) 10%, (b) 20%, (c) 25%, (d) 30%, (e) 40%, and (f) 50%].

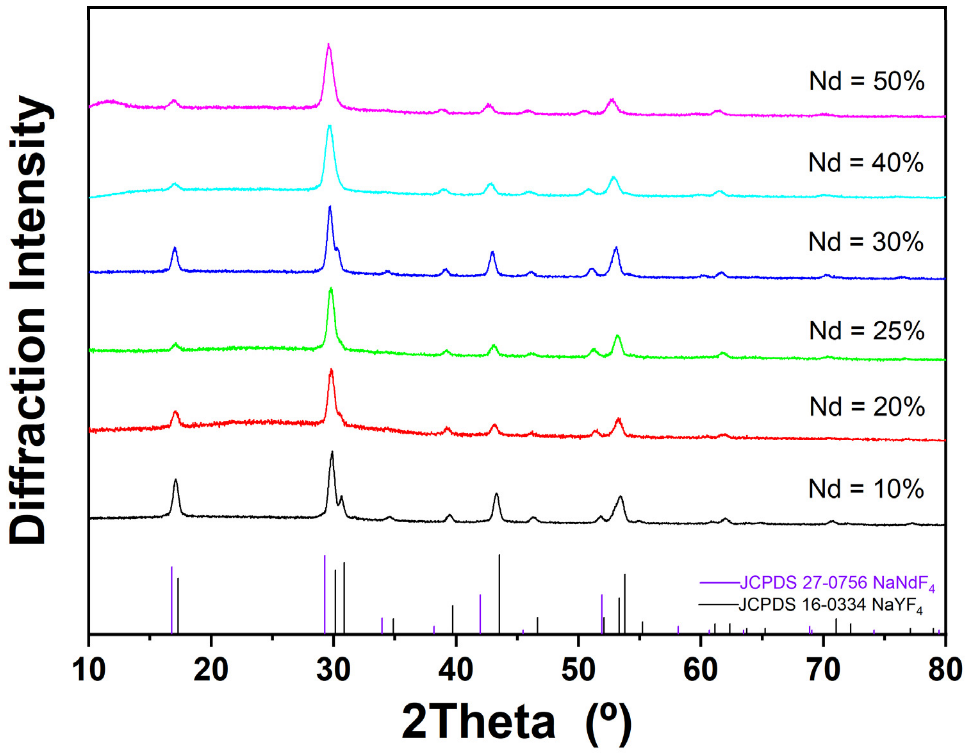

Figure S2. XRD patterns of $NaYF_4$:Nd(x%),Yb(10%) core DSNPs with various Nd^{3+} concentrations [x = 10, 20, 25, 30, 40, and 50%].

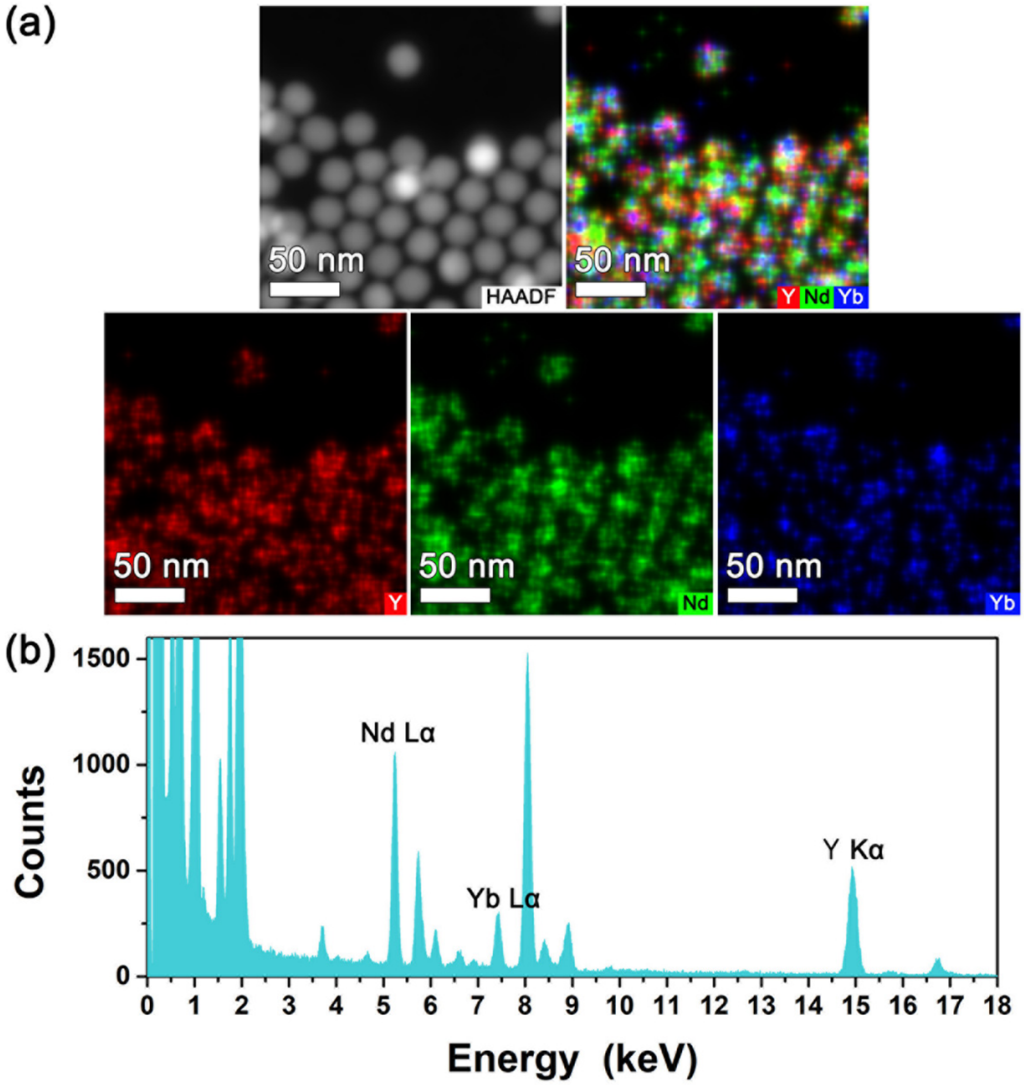

Figure S3. (a) HAADF-STEM image and EDS map images (merged image, Y Kα, Nd Lα, and Yb Lα map images) of $NaYF_4$:Nd(30%),Yb(10%) core DSNPs. (b) EDS spectrum of $NaYF_4$:Nd(30%),Yb(10%) core DSNPs.

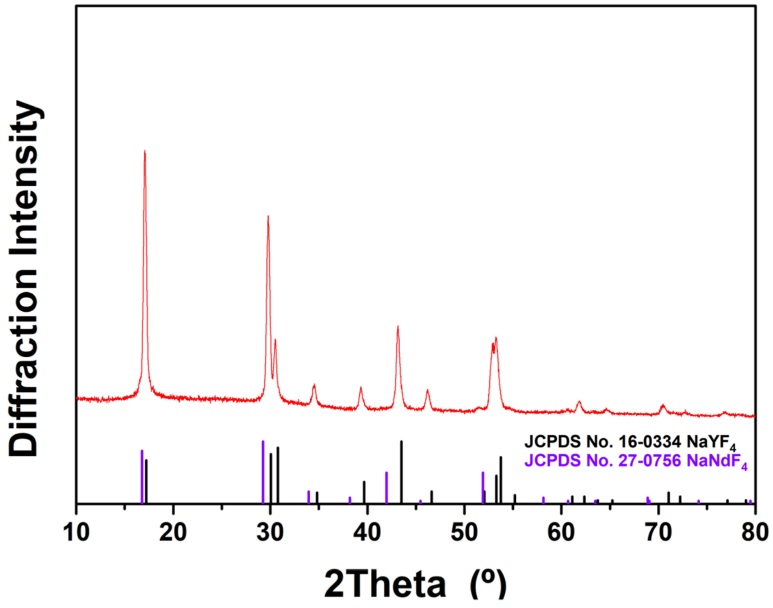

Figure S4. XRD pattern of $NaYF_4$:Nd(30%),Yb(10%)/$NaYF_4$:Nd(10%)/$NaYF_4$ C/S/S DSNPs.

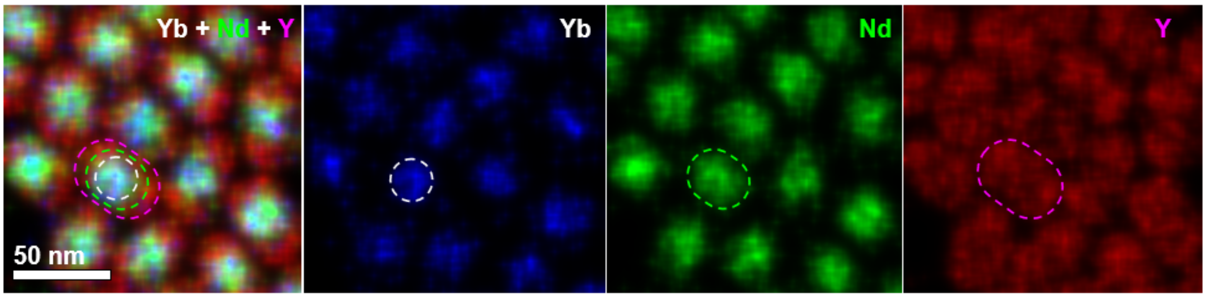

Figure S5. EDS map images showing the distribution of Yb (blue color), Nd (green color), and Y (red color) of the $NaYF_4$:Nd(30%),Yb(10%)/$NaYF_4$:Nd(10%)/$NaYF_4$ C/S/S DSNPs. The composite EDS map image clearly demonstrates that the synthesized DSNPs have core/shell/shell structure.

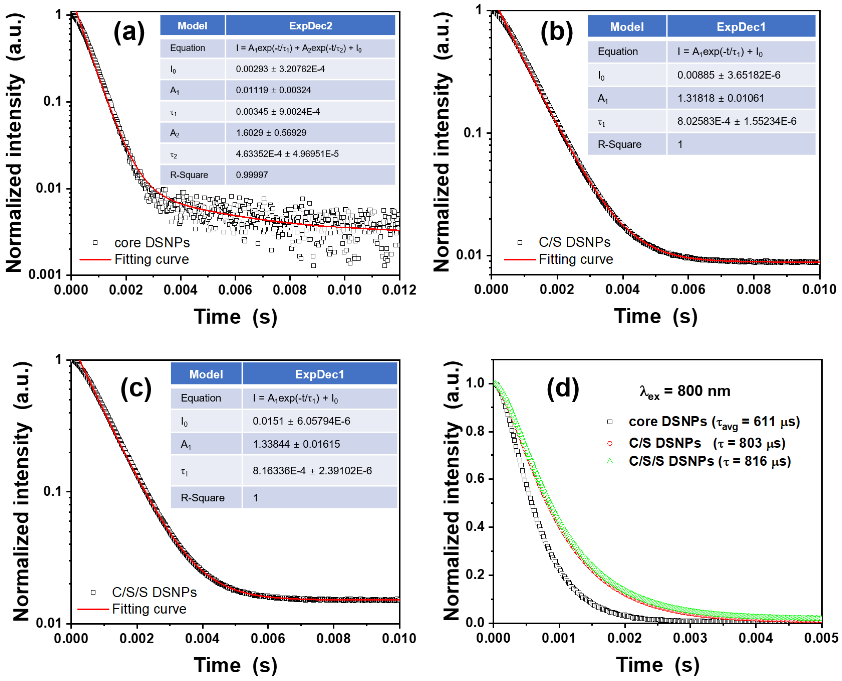

Figure S6. Time-resolved PL profiles with fitted curves of (a) $NaYF_4$:Nd(30%),Yb(10%) core DSNPs, (b) $NaYF_4$:Nd(30%),Yb(10%)/$NaYF_4$:Nd(10%) C/S DSNPs, and (c) $NaYF_4$:Nd(30%),Yb(10%)/$NaYF_4$:Nd(10%)/$NaYF_4$ C/S/S DSNPs under 800 nm excitation. (d) Time-resolved PL profiles of $NaYF_4$:Nd(30%),Yb(10%) core DSNPs (black square), $NaYF_4$:Nd(30%),Yb(10%)/$NaYF_4$:Nd(10%) C/S DSNPs, (red circle), and $NaYF_4$:Nd(30%),Yb(10%)/$NaYF_4$:Nd(10%)/$NaYF_4$ C/S/S DSNPs (green triangle). In the case of the core DSNPs, the average PL lifetime can be obtained by using a following Equation (S1) [S1],

$$\tau_{avg} = \frac{A_1\tau_1^2+A_2\tau_2^2}{A_1\tau_1+A_2\tau_2} \quad \text{------------------------} \quad (S1)$$

For the core DSNPs, the average PL lifetime (τ_{avg}) was calculated to be 611 μs.

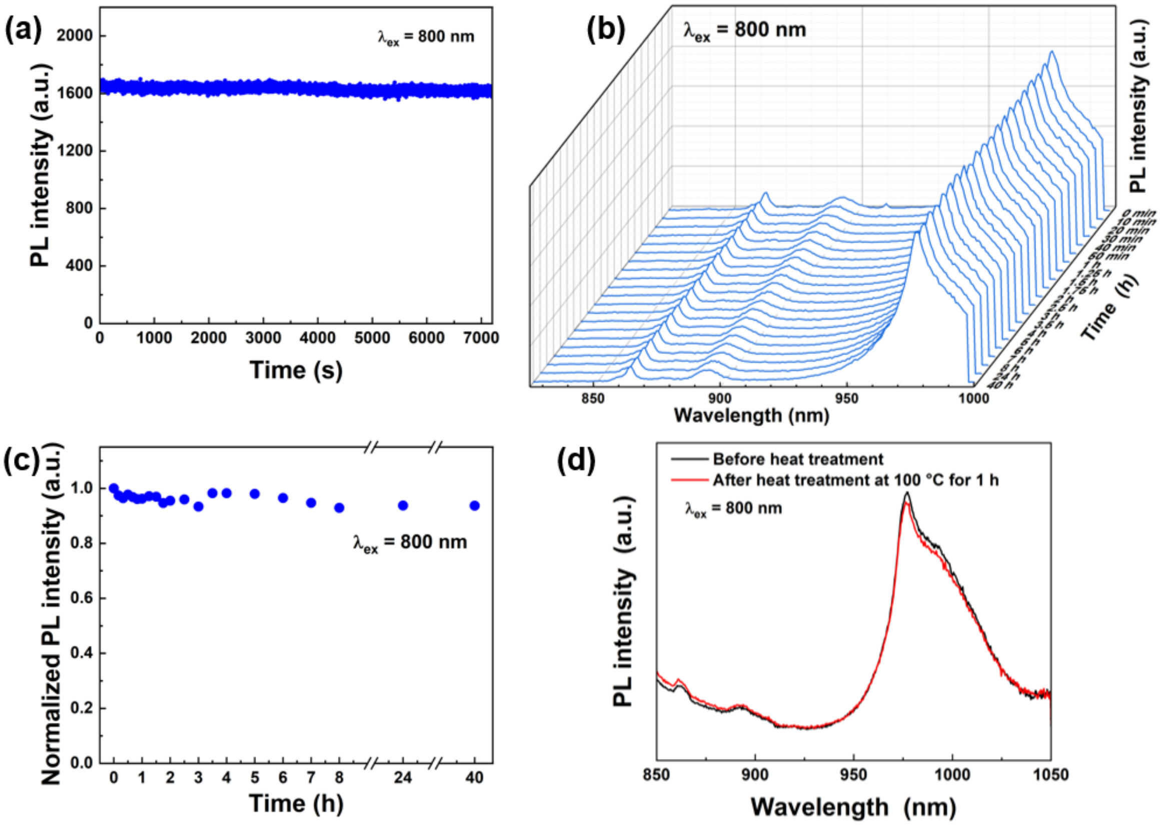

Figure S7. (a) PL intensities of the $NaYF_4$:Nd(30%),Yb(10%)/$NaYF_4$:Nd(10%)/$NaYF_4$ C/S/S DSNPs monitored at 978 nm under continuous irradiation with an 800 nm NIR laser (27.2 W·cm^{-2}). (b) PL spectra and (c) normalized PL intensities of the $NaYF_4$:Nd(30%),Yb(10%)/$NaYF_4$:Nd(10%)/$NaYF_4$ C/S/S DSNPs under irradiation with a 365 nm UV lamp (6 W). (d) PL spectra of the $NaYF_4$:Nd(30%),Yb(10%)/$NaYF_4$:Nd(10%)/$NaYF_4$ C/S/S DSNPs before and after heat treatment at 100 °C for 1 h.

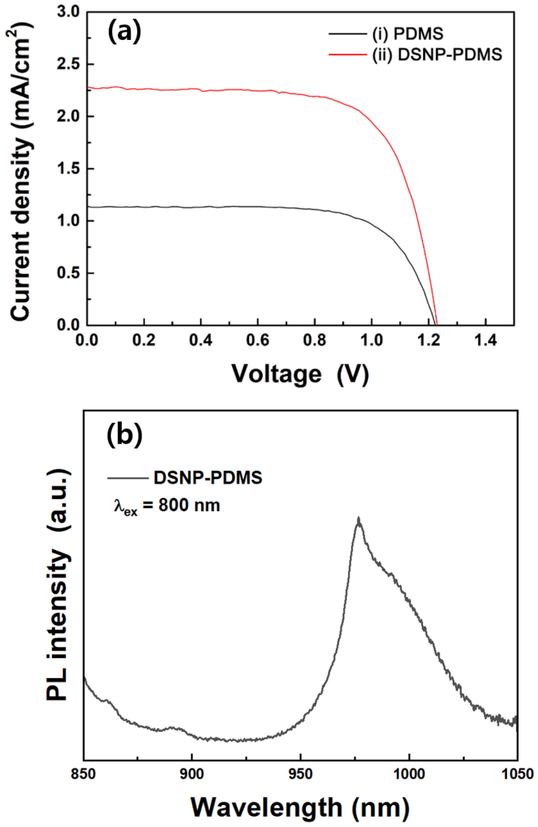

Figure S8. (a) Current density versus voltage curves of (i) bare PDMS- and (ii) C/S/S DSNP-PDMS composite-coupled silicon solar cells. (b) PL spectrum of the C/S/S DSNP-PDMS composite under excitation with 800 nm NIR light (1 mW).

Reference

[S1] Kıbrıslı, O.; Vahedigharehchopogh, N.; Ersundu, A. E.; Çelikbilek Ersundu, M. Instantaneous Color Tuning of Upconversion Emission in a Novel Lanthanide-Doped Monolithic Glass via Excitation Modulation. *J. Phys. Chem. C* **2020,** *124*, 10687-10695.
